# Does sinus membrane thickness influence the risk of perforation during lateral sinus lift surgery for dental implants? a systematic review and meta-analysis

**DOI:** 10.4317/medoral.26545

**Published:** 2024-05-25

**Authors:** Yunyan Ke, Guihong Xuan

**Affiliations:** 1 Department of Stomatology, Shaoxing Hospital of Traditional Chinese Medicine, Shaoxing City, Zhejiang Province, China; 2 Department of Stomatology, Shaoxing People's Hospital, Shaoxing City, Zhejiang Province, China

## Abstract

**Background:**

We reviewed the literature to examine if the thickness of the sinus membrane is a risk factor for perforation during lateral sinus lift surgery.

**Material and Methods:**

We searched Embase, PubMed, and Web of Science databases till 4th December 2023 for studies examining the risk of perforation with different sinus membrane thicknesses. Studies reporting sinus membrane thickness in perforation and non-perforation cases were also included.

**Results:**

Eleven studies were eligible. All studies used cone beam computed tomography for measuring sinus membrane thickness. Meta-analysis showed that sinus membrane thickness was significantly lower in perforation cases as compared to non-perforation cases (MD: -0.91 95% CI: -1.48, -0.33 I2=94%). Four studies used 2mm as the cut-off to define thick and thin sinus membranes. Pooled analysis failed to demonstrate any significant difference in perforation rates (OR: 0.97 95% CI: 0.44, 2.17 I2=56%). Meta-analysis of studies using 1.5mm (OR: 0.66 95% CI: 0.29, 1.48 I2=72%) and 1mm cut-off (OR: 0.93 95% CI: 0.34, 2.56) also demonstrated similar non-significant results.

**Conclusions:**

Our study shows that the sinus membrane is significantly thinner in cases with perforations as compared to those with no perforations. However, a meta-analysis based on different membrane thickness cut-offs failed to demonstrate a relationship between thinner sinus membranes and a higher risk of perforation. There is a need for further studies examining the role of sinus membrane thickness on perforation rates.

** Key words:**Schneiderian membrane, dental implants, lateral window, perforation, sinus augmentation.

## Introduction

Rehabilitation of an edentulous posterior maxilla has been challenging owing to the limited density of bone and proximity of the maxillary sinus. Loss of the teeth and age-related changes lead to resorption of the alveolar bone leaving minimal bone between the crest and the maxillary sinus which makes dental implant placement difficult (1). In such cases, implant rehabilitation can be done by augmenting the bone on the crest or elevating the sinus membrane to allow sufficient space and prevent protrusion of the implants in the maxillary sinus.

Maxillary sinus augmentation has become a standard procedure to augment bone in the posterior maxilla for dental implant rehabilitation (2). Two major techniques have been described, namely, the lateral window technique and the osteotome-mediated transalveolar sinus lift (3). The former is more commonly used as it allows for significant membrane elevation and bone grafting compared to the transalveolar method. In clinical practice, the lateral window approach is used when the residual bone height is <5mm (4). Research shows high success rates ranging from 88-100% in cases of implant placement with sinus augmentation using the lateral window approach (5,6).

Several factors can influence the success of sinus augmentation with the lateral window technique. The curvature of the sinus, presence of septae, bone height, and antroliths can alter the success of sinus augmentation (7,8). Perforation of the sinus membrane is considered an important intraoperative complication that can jeopardize the procedure and cause dislodgement of the graft into the sinus cavity (9). However, there is conflicting evidence on the effect of such perforations on the survival of dental implants with some studies reporting reduced survival rates while others reporting no impact on success/survival (10-12). Nevertheless, intraoperative sinus perforations require appropriate management techniques to accommodate the graft and improve the outcome of the procedure (13). Also, membrane perforations can disrupt the normal physiologic sinus function and cause postsurgical sinusitis (14). One important factor considered by several studies in literature as a risk factor for membrane perforation is membrane thickness. Nevertheless, there is no clarity on how membrane thickness influences the risk of perforation. We hereby reviewed the evidence in the literature to examine if sinus membrane thickness is a risk factor for perforation during sinus augmentation via the lateral window approach.

## Material and Methods

The current work is a systematic review and meta-analysis of observational studies reported in line with the PRIMA guidelines (15). The protocol was prepared and registered on the international database of systematic reviews, PROSPERO (accessible at https://www.crd.york.ac.uk/prospero/ with the registration number, CRD42023483303).

- Search and eligibility criteria

Articles were searched from the literature by two reviewers in collaboration with a medical librarian experienced in systematic reviews. English-language studies listed on the databases of Embase, PubMed, and Web of Science were searched based on the listed inclusion criteria. The search was completed on 4th December 2023. The search queries used are shown in Table 1 and include a combination of the keywords: “sinus membrane; Schneiderian membrane; sinus lift; sinus augmentation, thickness; complications; and perforation”. The search strategy combined these keywords into various combinations with “AND” and “OR” to optimize the results.

The results of the databases were then combined in the reference manager software. Any studies found by gray literature search were then added. All duplicate studies were removed and the remaining were scanned based on the inclusion criteria.

Inclusion criteria were: 1. Studies conducted on patients undergoing lateral window sinus lift 2. Studies comparing patients with thick vs thin sinus membranes and reporting perforation rate OR 3. Studies report sinus thickness in perforation and non-perforation cases.

Studies on osteotome sinus lift procedures, studies not reporting sinus thickness, and not reporting perforation rates were excluded. Conference abstracts and unpublished data were also not eligible for inclusion.

The reviewers identified relevant studies and read their full texts. Studies fulfilling all criteria were included. The reference list of these studies was also searched for any missed trials. Any disagreements between reviewers were resolved by discussion.

- Extracted data and outcomes

Data collected from the studies included: authors, publication year, study type, sample size, mean age, gender, residual bone height, number of patients with perforation, cut-off for sinus membrane thickness, and membrane thickness in perforation and non-perforation cases. Using a data extraction form, two reviewers collected the data. Any variations were then cross-checked and corrected by discussion. If data was incompletely reported, the Correspondence was contacted once by email.

- Evaluation of study quality

Two authors independently assessed methodological assessment for risk of bias. The Newcastle Ottawa Scale (NOS) (16) was used as all studies were observational. Points were awarded for the representativeness of the study cohort, comparability of groups, and measurement of outcomes with each receiving a maximum of four, two, and three points respectively.

- Statistical analysis

All statistical analysis was done on “Review Manager” (RevMan, version 5.3; Nordic Cochrane Centre (Cochrane Collaboration), Copenhagen, Denmark; 2014). Given the inherent methodological heterogeneity between studies, we preferred the random-effects meta-analysis model. Continuous and binary outcomes were pooled to generate mean difference (MD) and odds ratio (OR) respectively with 95% confidence intervals (CI). Funnel plots were drawn for publication bias. The I2 statistic was the tool to determine inter-study heterogeneity. I2 <50% meant low and >50% meant substantial heterogeneity. *P* values below 0.05 were considered significant. Subgroup analysis was conducted for different cut-offs of sinus membrane thickness. A leave-one-out meta-analysis was conducted to check for outliner studies.

## Results

**Figure 1:**
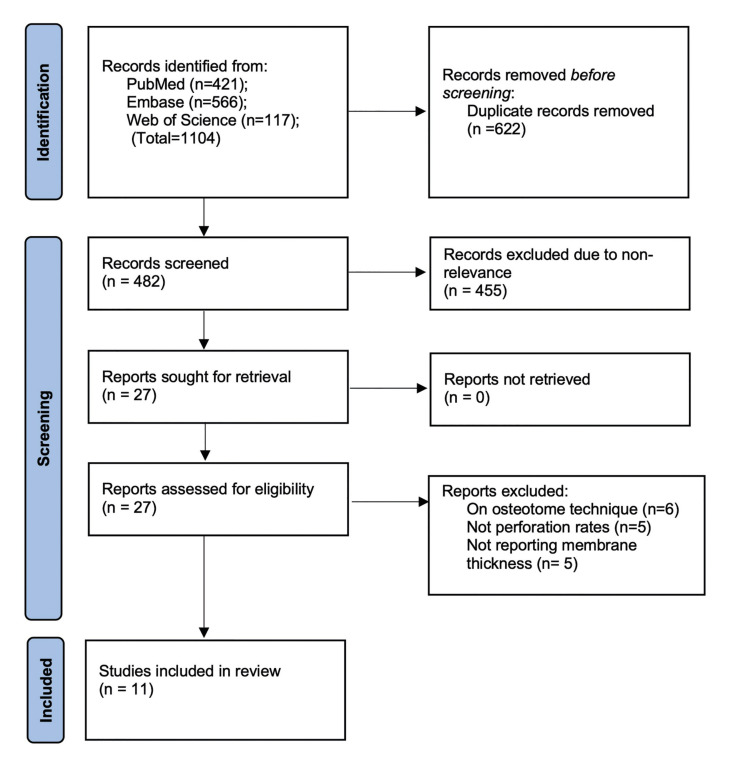
Flow-chart of the search.

The number of search results and study selection process are shown in the PRISMA flow diagram (Fig. 1). The literature search initially led to 1104 citations. After deduplication, 482 articles were screened and 27 were found relevant to the review of which eleven were selected (17-27). The agreement between the reviewers was high (kappa=0.95).

Details of the eleven studies are shown in Table 2. The year of publication of studies ranged from 2014 to 2023. Two were prospective while others were retrospective. Studies were from China, Iran, Austria, the USA, Korea, Taiwan, Turkey, and Switzerland. Cumulatively 1841 sinus lift procedures were examined in the included studies. The mean age ranged from 46.3 years to 57 years.

All studies used preoperative cone beam computed tomography (CBCT) to measure the sinus membrane thickness. The perforation rate in studies ranged from 10 to 37%. None of the studies used propensity score matching to match the baseline groups, hence the NOS score was 7 for all studies.

**Figure 2:**
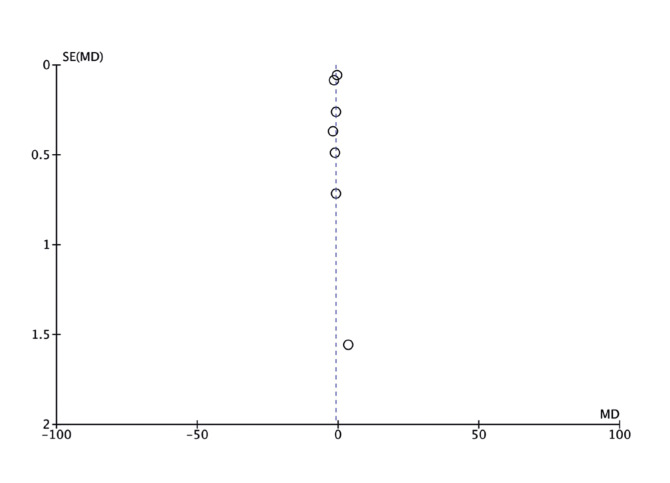
Meta-analysis of sinus membrane thickness in perforation and non-perforation cases.

**Figure 3:**
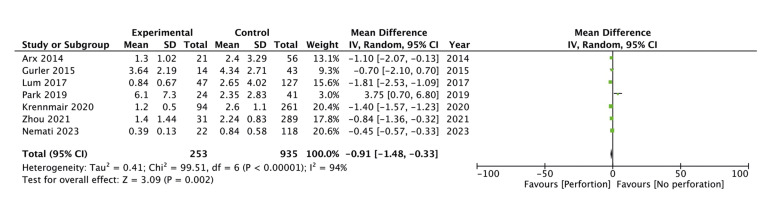
Funnel plot to visualize publication bias.

Seven studies reported sinus membrane thickness in perforation and non-perforation cases. Meta-analysis showed that sinus membrane thickness was significantly lower in perforation cases as compared to non-perforation cases (MD: -0.91 95% CI: -1.48, -0.33 I2=94%) (Fig. 2). On leave-one-out analysis, no study was found to be an outliner and the results did not change in statistical significance. The funnel plot showed no gross asymmetry indicating no publication bias (Fig. 3).

**Figure 4:**
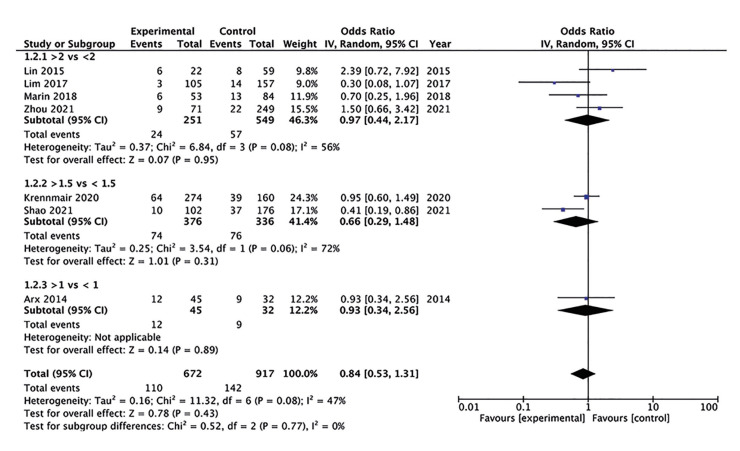
Meta-analysis of perforation rates with different sinus membrane thickness cut-offs.

Meta-analysis based on various sinus membrane thickness cut-offs is shown in Fig. 4. Four studies used 2mm as the cut-off to define thick and thin sinus membranes. Pooled analysis failed to demonstrate any significant difference in perforation rates (OR: 0.97 95% CI: 0.44, 2.17 I2=56%). Meta-analysis of studies using 1.5mm (OR: 0.66 95% CI: 0.29, 1.48 I2=72%) and 1mm cut-off (OR: 0.93 95% CI: 0.34, 2.56) also demonstrated similar non-significant results (Fig. 4).

## Discussion

The most important structure involved in sinus augmentation surgery is the Schneiderian membrane itself which needs to be elevated from the maxillary bone. The membrane consists of three layers namely, pseudostratified columnar epithelium, a vascular lamina propria, and the periosteum. Prior anatomic studies have shown that mean sinus membrane thickness is about 0.09mm ranging from 0.02 to 0.35mm (28) while clinical studies report a thickness of usually 1-2mm (29,30). Membrane perforation is one of the most common complications seen in approximately 30% of all sinus lift cases (31). Perforation of the membrane can occur at different steps of the procedure like preparation of the bony window, elevation of the lining, placement of graft material, and placement of the implant itself. Advances in technology and the adoption of piezoelectric devices replacing conventional rotary instruments have helped reduce the risk of perforations during the initial step (32). Nevertheless, such perforations are not completely avoidable and often lead to sinus pathologies and also affect graft and implant survival rates (10-12). Therefore, it is prudent that factors influencing membrane perforation are recognized and such complications avoided to improve the success of the surgery (29,30).

An in-vitro study by Pommer *et al* (28) on 20 cadavers has shown that the sinus membrane can be stretched to 125 to 133% of its original size in one and two dimensions respectively. Also, force greater than 7.3N/mm2 led to membrane perforations with thicker membranes showing higher load limits. In clinical practice, sinus membrane thickness of <2mm is considered normal while those with more than 2mm thickness are considered pathological (29). Pathological sinus thickening is not uncommon and is noted in around 12-30% of patients reporting for dental treatment (33,34). In this meta-analysis, we thoroughly examined the association between the thickness of the sinus membrane and the risk of perforations during the procedure. In the first analysis, it was noted that sinus membrane thickness in perforation cases was significantly lower as compared to non-perforation cases. The results were consistent amongst the included studies with six of the seven studies in the meta-analysis noting thinner membranes in cases with perforation. However, on segregating the patients based on a specific cut-off of membrane thickness, we could not find any statistically significant results. There was no difference in perforation rates based on cut-offs of 2mm, 1.5mm and 1mm. One reason for such inconsistent results could be the lower number of studies in the later meta-analysis and the variability in the cut-offs used by the included studies. Secondly, Lin *et al* (21) have shown that the relationship between membrane thickness and perforation follows a U-shaped curve. A higher risk of perforations was noted in both very thin and thick sinuses. In their study, cases with sinus membrane thickness of ≤0.5mm and >3mm had perforation rates of 17% and 25% respectively and the lowest perforation rate of 7% was seen with a thickness of 1-1.5mm. Thus, it is plausible that very thin sinus membranes have lower load limits causing a higher number of perforations. However, even extremely thick pathological membranes are not amenable to sinus augmentation procedures and the thickened connective tissue does not have a similar load limit as that of a healthy membrane.

Prior reviews have also shown reduced membrane perforations with thickened sinuses. Monje *et al* (30) in a meta-analysis of six studies noted no statistical difference in the perforation rates with thick and thin sinus linings but showed a reduced trend of perforations with thicker membranes. In comparison with the current review, Monje *et al* (30) did not perform a subgroup analysis of different cut-offs of membrane thickness and all studies with different cut-offs were pooled in a single analysis. Another review by Fang *et al* (29) combined studies on both the lateral window and osteotome technique to show lower perforation rates in patients with thickened sinuses. The perforation rate in normal sinuses was 14% and in cases with thick sinus lining, it was 6%. By conducting an updated literature search and including maximum studies on a single technique of sinus augmentation, our review presents the most comprehensive and up-to-date evidence on the association between sinus membrane thickness and the risk of perforations with the lateral window technique. Additionally, two separate meta-analyses were conducted in our review. One on the thickness of the membrane in perforation and non-perforation cases and the second using different cut-offs of membrane thickness.

Nevertheless, there are limitations to this study. The number of studies in each meta-analysis was not high. Inconsistent cut-offs and reporting of data precluded a meta-analysis of all 11 studies in both meta-analyses. Secondly, sinus membrane perforation is also dependent on several other confounding factors like the size of the edentulous region, the thickness of the lateral window, the type of instruments used, the presence of septae, the amount of bone graft inserted, etc. Additionally, the experience of the operator can also impact the risk of complications. It is necessary to understand that the current results are based on crude data and several such known and unknown confounders could have altered the risk of perforations. Thirdly, the size of the perforations was not accounted for in the included studies. Perforation size has important intra-operative and prognostic significance as small perforations can be left in situ or covered with a collagen membrane while extremely large perforations may lead to abandoning of the procedure (9). Lastly, all studies used CBCT to measure the thickness of the sinus membrane and it may not be an accurate measurement as compared to histological thickness.

## Conclusions

Our study shows that the sinus membrane is significantly thinner in cases with perforations as compared to those with no perforations. However, a meta-analysis based on different membrane thickness cut-offs failed to demonstrate a relationship between thinner sinus membranes and a higher risk of perforation. There is a need for further studies examining the role of sinus membrane thickness on perforation rates.

## Figures and Tables

**Table 1:** Search queries.

Search queries
(((sinus lift) OR (sinus augmentation)) AND (thickness)) AND (complications)
(((schneiderian membrane) OR (sinus membrane)) AND (thickness)) AND (complications)
(((sinus lift) OR (sinus augmentation)) AND (thickness)) AND (perforation)
(((schneiderian membrane) OR (sinus membrane)) AND (thickness)) AND (perforation)

**Table 2:** Data extracted from included studies.

Study	Location	Type	Sample size (sinuses)	Mean age (years)	Mean bone height (mm)	Perforation	Membrane thickness	Cut-off	Perforation rate	NOS score
Nemati 2023 (27)	Iran	P	140	54.6	2.83	Yes: 22 No: 118	0.39 ± 0.13 0.84 ± 0.58	NR	NR	7
Zhou 2021 (26)	China	R	320	51.5	NR	Yes: 31 No: 289	1.40± 1.44 2.24± 0.83	<2: 249 >2: 71	22 9	7
Shao 2021 (17)	China	R	278	50.3	3.79	Yes: 47 No: 231	NR	<1.5: 176 >1.5: 102	37 10	7
Krennmair 2020 (25)	Austria	R	355	55.9	3.89	Yes: 94 No: 261	1.2± 0.5 2.6± 1.1	<1.5: 160 >1.5: 274	39 64	7
Park 2019 (24)	USA	R	65	59	NR	Yes: 24 No: 41	6.1± 7.3 2.35± 2.83	NR	NR	7
Marin 2018 (22)	Austria	P	137	55.1	NR	Yes: 19 No: 118	NR	<2: 84 >2: 53	13 6	7
Lum 2017 (23)	USA	R	167	58.5	3.21	Yes: 47 No: 127	0.84± 0.67 2.65± 4.02	NR	NR	7
Lim 2017 (20)	Korea	R	172	46.3	NR	Yes: 17 No: 155	NR	<2: 157 >2: 105	14 3	7
Lin 2015 (21)	Taiwan	R	73	53.8	3.07	Yes: 14 No: 59	NR	<2: 59 >2: 22	8 6	7
Gurler 2015 (19)	Turkey	R	57	49.6	NR	Yes: 14 No: 43	3.64± 2.19 4.34± 2.71	NR	NR	7
Arx 2014(18)sex, smoking habit	Switzerland	R	77	57	NR	Yes: 21 No: 56	1.3± 1.02 2.4± 3.29	<1: 32 >1: 45	9 12	7

CBCT, cone beam computed tomography; NR, not reported; Newcastle Ottawa score.
